# The unrecognized burden of cardiovascular risk factors in women newly diagnosed with endometrial cancer: A prospective case control study

**DOI:** 10.1016/j.ygyno.2017.11.019

**Published:** 2018-01

**Authors:** Sarah J. Kitson, Jennifer Lindsay, Vanitha N. Sivalingam, Mark Lunt, Neil A.J. Ryan, Richard J. Edmondson, Martin K. Rutter, Emma J. Crosbie

**Affiliations:** a Division of Cancer Sciences, Faculty of Biology, Medicine and Health, University of Manchester, St Mary's Hospital, Manchester M13 9WL, United Kingdom; b Division of Musculoskeletal and Dermatological Sciences, University of Manchester, Manchester M13 9PL, United Kingdom; c Department of Obstetrics and Gynaecology, Manchester University NHS Foundation Trust, Manchester Academic Health Science Centre, Manchester M13 9WL, United Kingdom; d Division of Diabetes, Endocrinology and Gastroenterology, School of Medical Sciences, Faculty of Biology, Medicine and Health, University of Manchester, Manchester M13 9PL, United Kingdom; e Manchester Diabetes Centre, 193 Hathersage Road, Manchester University NHS Foundation Trust, Manchester Academic Health Science Centre, Manchester M13 0JE, United Kingdom

**Keywords:** Endometrial cancer, Obesity, Hyperglycemia, Hypertension, Hypercholesterolemia, Cardiovascular disease, QRISK2, Survival

## Abstract

**Background:**

Cardiovascular disease is a major cause of death in endometrial cancer survivors. The aim of this study was to determine whether women newly diagnosed with endometrial cancer have a higher prevalence of cardiovascular risk factors than the general population.

**Methods:**

The prevalence of adequately treated and unrecognized/inadequately treated cardiovascular risk factors and the corresponding 10-year cardiovascular risk by QRISK2 score was measured in 150 consecutive women undergoing primary treatment for endometrioid endometrial cancer in the North West of England, and 746 age and ethnicity-matched control women from the Health Survey for England 2014.

**Results:**

Women with endometrial cancer had higher proportions of obesity (BMI ≥ 30 60.7% vs. 32.4%, p < 0.0001) and a preponderance of unrecognized and inadequately treated cardiovascular risk factors. Compared with controls, endometrial cancer cases had a higher prevalence of incident hyperglycemia (57.2% vs. 11.5%, p < 0.0001), total: HDL cholesterol ratio > 4.5 (26.7% vs. 13.7%, p < 0.0001), and were more likely to have three or more cardiovascular risk factors (22% vs. 6%, p < 0.0001). This equates to a higher 10-year cardiovascular risk (median QRISK2 score 12.6% vs. 8.8%, p < 0.0001). Optimization of risk factors would have a greater impact on absolute cardiovascular disease risk for cases than controls (QRISK2 score reduction 1.8% vs. 0.7%).

**Conclusions:**

Women undergoing primary treatment for endometrial cancer have a higher prevalence of cardiovascular risk factors than women without the disease. Early identification and treatment of these risk factors could improve outcomes for endometrial cancer survivors.

## Highlights

• CVD risk was measured in a prospective study of 150 EC patients and 746 controls.
• EC patients had significantly more CVD risk factors than other women.
• Most CVD risk factors were either unrecognized or inadequately treated.
• EC patients had a significantly higher 10-year risk of CVD (QRISK2) than controls.
• Identifying and treating CVD risk factors could improve outcomes for EC survivors.

## 1 Introduction

Endometrial cancer, the fourth most common female malignancy, affects over 9000 women each year in the UK and its incidence is rising, such that by 2030 it is estimated that there will be an additional 3600 new cases diagnosed every year in England and Wales alone [1], [2], [3]. Similar trends have been reported in other countries, for example in the USA, where 60,000 women are diagnosed annually and endometrial cancer is set to overtake lung and colorectal cancer to become the third most common female malignancy [4], [5]. Endometrioid endometrial cancer accounts for 80% of cases and its early presentation with postmenopausal bleeding means that the majority of women are diagnosed with disease confined to the uterus, making it amenable to curative surgical resection [6]. More women than ever are thus surviving a diagnosis of endometrial cancer, with 79% and 77.5% of women expected to live at least five and 10 years respectively following diagnosis [7].

Despite this, women with a history of endometrial cancer have a higher mortality rate than the general population, particularly if diagnosed at a young age [8]. Rather than dying of their endometrial cancer, however, cardiovascular deaths predominate in those with early stage disease and this becomes more pronounced the longer women survive their cancer diagnosis. Overall, the risk of death from myocardial infarction and stroke is estimated to be two-fold higher than the risk of death from endometrial cancer and nearly nine-fold higher than that of women from the general population [8], [9].

This is not surprising given that endometrial cancer and cardiovascular disease share the common risk factors of obesity and diabetes [10]. Obesity is the strongest risk factor for endometrial cancer, driving carcinogenesis through unopposed estrogen excess, hyperinsulinemia and insulin resistance [2], [11], [12]. Women with diabetes have a two-fold higher risk of endometrial cancer compared with non-diabetic women, even after adjustment for body mass index (BMI), suggesting an independent relationship between the two conditions.

Yet screening for, and optimization of, cardiovascular risk factors is not routinely undertaken in endometrial cancer survivors. Indeed, the true prevalence of hypercholesterolemia, hypertension and hyperglycemia in women with endometrial cancer is unknown. Previous estimates have been based on established diagnoses of cardiovascular risk factors, which may underestimate the true prevalence of conditions that are often asymptomatic [13], [14], [15]. This makes it difficult to estimate the benefit to be gained from introducing a program of routine testing and treatment of cardiovascular risk factors in endometrial cancer survivors.

In the current study, we asked whether women newly diagnosed with endometrial cancer have a higher prevalence of known and unrecognized cardiovascular risk factors than the general female population. Using the QRISK2 score, a widely-used UK-based validated cardiovascular risk calculator [16], we estimated the 10-year risk of cardiovascular disease in the two groups and calculated the likely benefit to be derived from optimization of modifiable risk factors.

## 2 Methods

### 2.1 Study design

This was a prospective case-control study performed between 2016 and 2017 in the North West of England.

### 2.2 Research ethics

The study was approved by the West of Scotland Research Ethics Committee (reference 16/WS/0040) and was prospectively registered on the NIHR Clinical Research Network Portfolio.

### 2.3 Selection of cases and data collection

We recruited consecutive patients with newly diagnosed endometrioid endometrial cancer referred for primary treatment by hysterectomy, who provided written, informed consent to participate in the study. A detailed medical history was obtained through interview and checked against medical records regarding known diagnoses of diabetes, pre-diabetic hyperglycemia, hypertension, hypercholesterolemia and cardiovascular disease, defined as a previous myocardial infarction, angina, coronary artery bypass graft, stroke or transient ischemic attack. Current medications used for the aforementioned conditions were also considered evidence of a prior diagnosis. Smoking status categorised women as never smokers, ex-smokers or current smokers and the number of cigarettes smoked per day was recorded.

Anthropometric measurements were recorded in a standard fashion; height was determined using a stadiometer and performed barefooted and weight measured using electronic scales after removal of bulky clothing. BMI was calculated using the formula weight (kg)/height (m)^2^. Venepuncture was performed after an overnight fast of at least six hours duration and blood sent to the Clinical Biochemistry Department of the Manchester University NHS Foundation Trust for routine analysis. Determination of glycosylated hemoglobin (HbA1C) was undertaken using high performance liquid chromatography whilst total and high density lipoprotein (HDL) cholesterol levels were measured using an enzymatic colorimetric method, all according to standard operating procedures. Measurement of blood pressure was performed at rest in a seated position using a calibrated, automated sphygmomanometer.

### 2.4 Selection of controls and data collection

Each endometrial cancer case was matched for age (± 5 years), female sex and ethnicity to five participants in the Health Survey for England (HSE) 2014, the details of which have been previously published [17]. In brief, the survey is performed annually and collects information on the general health of 8000 adults randomly selected by postcode from across England using standardised questionnaires. Participants are representative of the general population, with each region proportionally sampled in a similar age distribution to the wider UK population [17]. In particular, the prevalence of overweight and obese individuals are equivalent to whole population estimates (HSE 58% vs. National Statistics 58%) [17], [18]. Individual level data is made freely available through the NHS Digital website. Information on previous medical history, in particular a prior diagnosis of cardiovascular disease, hypertension and diabetes, and drug history is available. Weight and height measurements and fasted serum levels of total and HDL cholesterol and HbA1C are measured in a comparable way to that of cases and recorded on line. Limited information was available in the Health Survey for England on cancer status, in particular a prior history of endometrial cancer, and participants were therefore not excluded on this basis.

### 2.5 Outcome definitions

New diagnoses of non-diabetic hyperglycemia and type 2 diabetes were defined as an HbA1C between 42 and 48 mmol/mol and > 48 mmol/mol, respectively, in a woman not previously diagnosed with these conditions. The HbA1C values used are in accordance with recommendations from the World Health Organisation [WHO, [19]].

A new diagnosis of hypercholesterolemia was defined as a total:HDL cholesterol ratio > 4.5 in a woman not previously prescribed treatment with statins [20]. Inadequately treated hypercholesterolemia was defined as a total:HDL cholesterol ratio > 4.5 in a woman already taking statin therapy.

Newly diagnosed hypertension was defined as a systolic blood pressure of > 140 mm Hg in a person not previously known to have a physician-obtained diagnosis of hypertension, or taking antihypertensive therapy [16]. Inadequately treated hypertension was the persistence of a systolic blood pressure greater than this threshold in someone already taking antihypertensive medication.

### 2.6 QRISK2 score

The QRISK2 score was calculated using the validated 2016 version of the online calculator available at https://www.qrisk.org/2016/. QRISK2 derives cardiovascular disease risk estimates based on prospective data from the UK primary care population. It is therefore infrequently used in other countries because risk estimates may not be appropriately calibrated outside of the UK. Data were input on ethnicity, gender, smoking status, diabetic status, antihypertensive treatment, total:HDL cholesterol, systolic blood pressure, height and weight. Missing data were imputed by the calculator by substituting gender and aged-based average values. Data on co-morbidities, including atrial fibrillation, renal disease and rheumatoid arthritis, family history of cardiovascular disease and postcode were unavailable for the Health Survey for England cohort and so were not included in the final analysis.

The predicted 10-year cardiovascular disease risk for patients without pre-existing cardiovascular disease was compared with an optimized risk for that individual, based on the treatment of underlying modifiable risk factors (i.e. quitting smoking, systolic blood pressure reduced to 140 mm Hg, total:HDL ratio decreased to 4.5, BMI reduced to 25 kg/m^2^). The absolute change in predicted risk through the optimization of risk factors was calculated by subtracting the optimized risk from the predicted risk prior to risk factor optimization [21].

### 2.7 Statistical analysis

A power calculation was performed based on the median age of women with endometrial cancer in our study. We assumed that 22.9% of women aged 65 years would have a QRISK2 score of > 20% [6], [22]. To detect a two-fold difference in QRISK2 score in women with endometrial cancer compared to those without the disease with 90% power, 5% error and five matched controls per case, we calculated that 124 cases and 620 controls would be required.

Data are reported as median and interquartile ranges due to their non-parametric distribution. Groups were compared using the Mann-U Whitney test for continuous data and χ^2^ and Fisher's exact tests for categorical data.

A p value of ≤ 0.05 was considered statistically significant. All statistical analysis was conducted using SPSS version 23 and Graph Pad Prism 7.

## 3 Results

### 3.1 Description of cases and controls

One hundred and fifty women with endometrioid endometrial cancer (hereafter referred to as endometrial cancer, n = 144) or its precursor lesion, atypical endometrial hyperplasia (n = 6), were recruited. Eight-nine percent of women with endometrial cancer had early stage disease (stages I and II) at presentation, reflecting the stage distribution seen in the general endometrial cancer population in the UK.

Cases were matched with 746 controls from the Health Survey for England (2014) for age and ethnic background. There were insufficient female participants aged 75 years and over and of non-white ethnic backgrounds included in the survey for all cases to be matched with five controls. There were no missing data for cases and 2% missing data for Health Survey for England controls. Missing data was restricted to absent total and HDL cholesterol levels (1.9% of controls), HbA1C levels (2.1% of controls), blood pressure measurements (1.2% of controls) and information on statin use (0.5% of controls).

The demographic details of cases and controls are shown in Table 1. Whilst there was no difference in the proportion of women who were current or ex-smokers in the two groups, women with endometrial cancer had significantly higher BMIs than those without the disease, reflecting the strong association between endometrial cancer and obesity (BMI ≥ 30 60.7% cases vs. 32.4% controls, p < 0.0001). In contrast, the proportion of cases already diagnosed with cardiovascular disease was significantly lower than that seen in the control group (6.0% cases vs. 15.7% controls, p = 0.002).

**Table 1:** Demographic data for cases and controls.

Characteristic	Cases (n = 150)	Controls (n = 746)	p value
Age, median yrs. (IQR)	65 (57–72)	64 (54–71)	0.093
BMI, median kg/m^2^ (IQR)	32.5 (26.9–38.8)	27.2 (24.0–31.5)	< 0.0001⁎⁎⁎⁎
< 25	25 (16.7)	241 (32.3)	
25–29.9	34 (22.7)	258 (34.6)	
30–34.9	37 (24.7)	152 (20.4)	
35–39.9	20 (13.3)	64 (8.6)	
≥ 40	34 (22.7)	25 (3.4)	
Missing data	0 (0.0)	6 (0.8)	
Ethnicity, n (%)			0.081
White	137 (91.3)	680 (91.1)	
Indian	6 (4.0)	29 (3.9)	
Pakistani	4 (2.7)	20 (2.7)	
Black/African/Caribbean	3 (2.0)	17 (2.3)	
Smoking status, n (%)			0.271
Never smoked	88 (58.7)	394 (52.8)	
Ex-smoker	43 (28.7)	265 (35.5)	
Current smoker	19 (12.7)	87 (11.7)	
Diagnosed cardiovascular disease, n (%)			0.002⁎⁎
No	141 (94.0)	629 (84.3)	
Yes	9 (6.0)	117 (15.7)	

*** p < 0.001.

⁎⁎ p < 0.01.

⁎⁎⁎⁎ p < 0.0001.

### 3.2 Prevalence of known and screen-detected cardiovascular risk factors in cases and controls

**Fig. 1:**
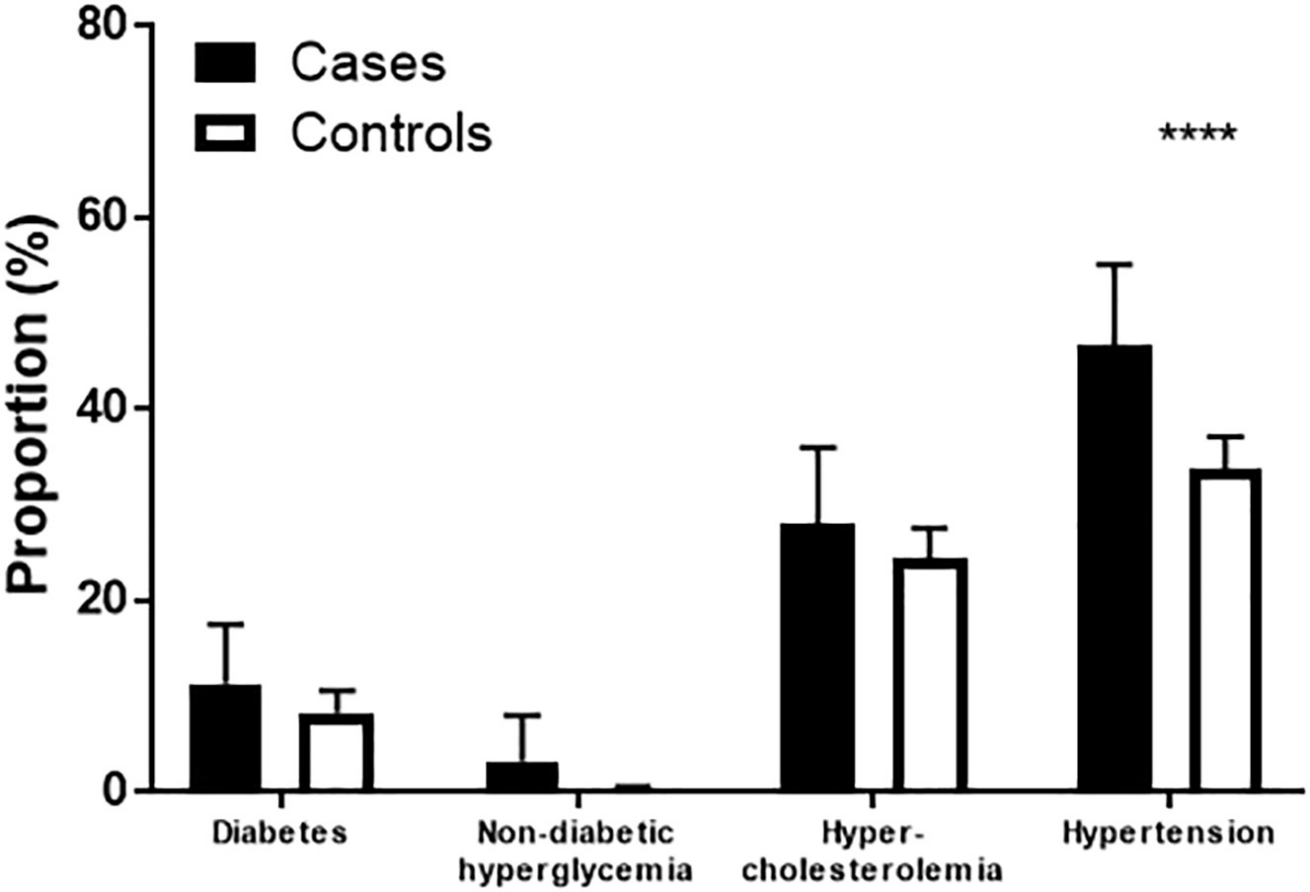
Prevalence of known individual cardiovascular risk factors in cases and controls. The proportion of women with known diabetes and hypercholesterolemia was similar in the two groups. A higher proportion of endometrial cancer survivors were already receiving treatment for hypertension than women in the general population (46.7% vs. 29.8%, p < 0.0001). As known diagnoses of non-diabetic hyperglycemia were not recorded in the Health Survey for England, comparisons of the prevalence of the condition between cases and controls was not possible. ****p < 0.0001

The prevalence of physician-diagnosed cardiovascular risk factors, specifically diabetes and hypercholesterolemia, was similar in the case and control groups (Fig. 1). Significantly more women with endometrial cancer, however, were likely to be taking antihypertensives than those in the general population (46.7% cases vs. 29.8% controls, p < 0.0001). Non-diabetic hyperglycemia had been previously diagnosed in 3.2% of cases. It was not recorded as a diagnosis in the Health Survey for England data, thereby preventing comparison of its prevalence between the two groups.

**Fig. 2:**
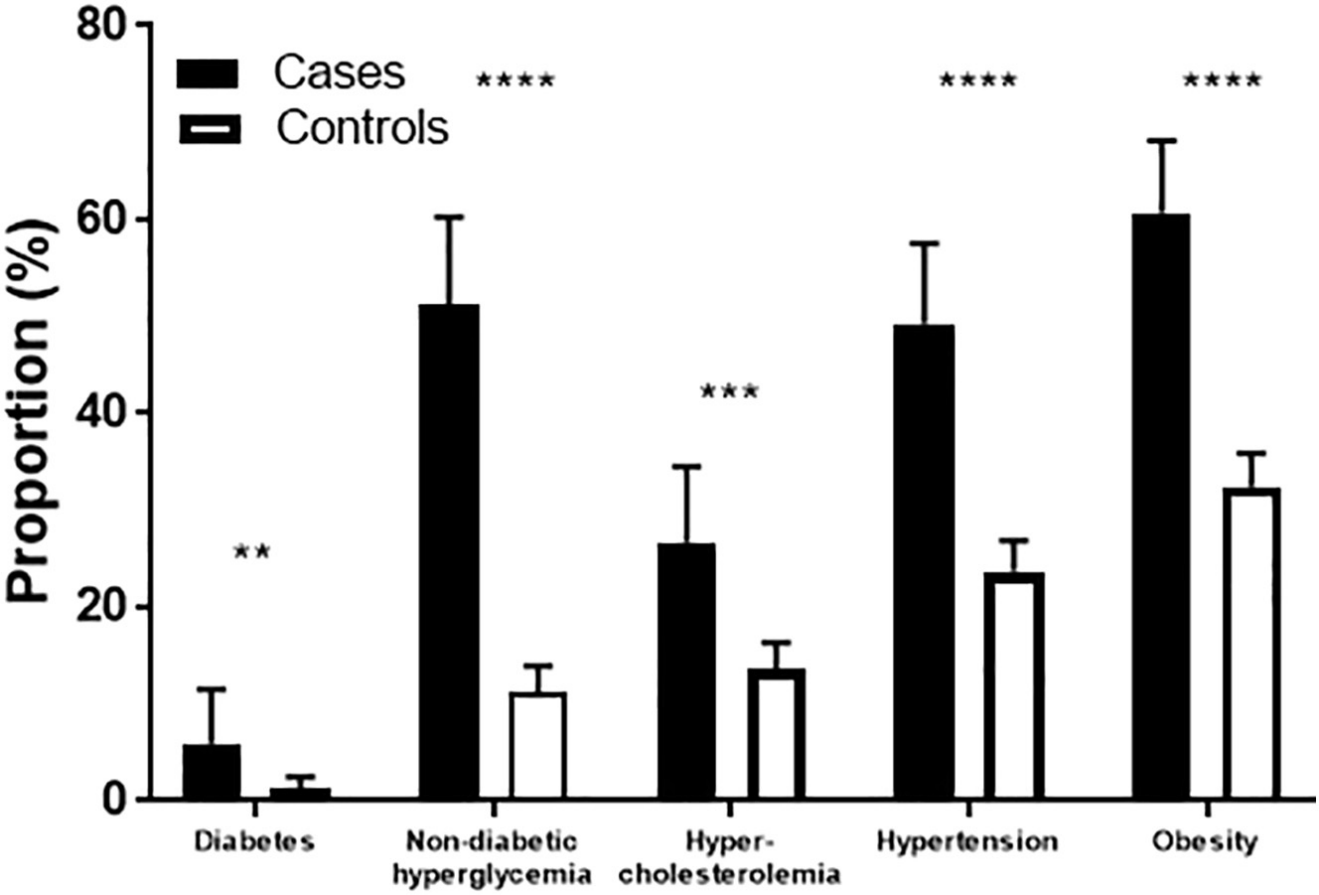
Prevalence of screen detected and undertreated individual cardiovascular risk factors in cases and controls. The prevalence of all of the cardiovascular risk factors studied was significantly higher in women undergoing treatment for endometrial cancer than the general population when screen detected and undertreated conditions were considered. Of particular note was that 57.2% of endometrial cancer survivors were found to have either diabetes or hyperglycemia that had been previously unrecognized compared with 11.5% of controls (p < 0.0001). **p ≤ 0.01, ***p ≤ 0.001.

In contrast to the equivalent proportions of known cardiovascular risk factors in the two populations, the prevalence of screen detected and undertreated risk factors was significantly higher in women with endometrial cancer than those without the disease (Fig. 2). A new diagnosis of diabetes and non-diabetic hyperglycemia was made according to elevated HbA1C values in 6.0% and 51.2% of cases compared with 1.3% and 10.2% of controls, respectively (p < 0.0001). Similarly, over a quarter of cases had an elevated total:HDL cholesterol ratio > 4.5, either in the absence of or despite statin therapy, compared with less than one in seven women in the control group (26.7% cases vs. 13.7% controls, p = 0.0002). Despite the higher proportion of women with endometrial cancer already receiving treatment for hypertension, more than twice as many cases had a systolic blood pressure > 140 mm Hg than controls (49.3% cases vs. 23.7% controls, p < 0.0001).

**Fig. 3:**
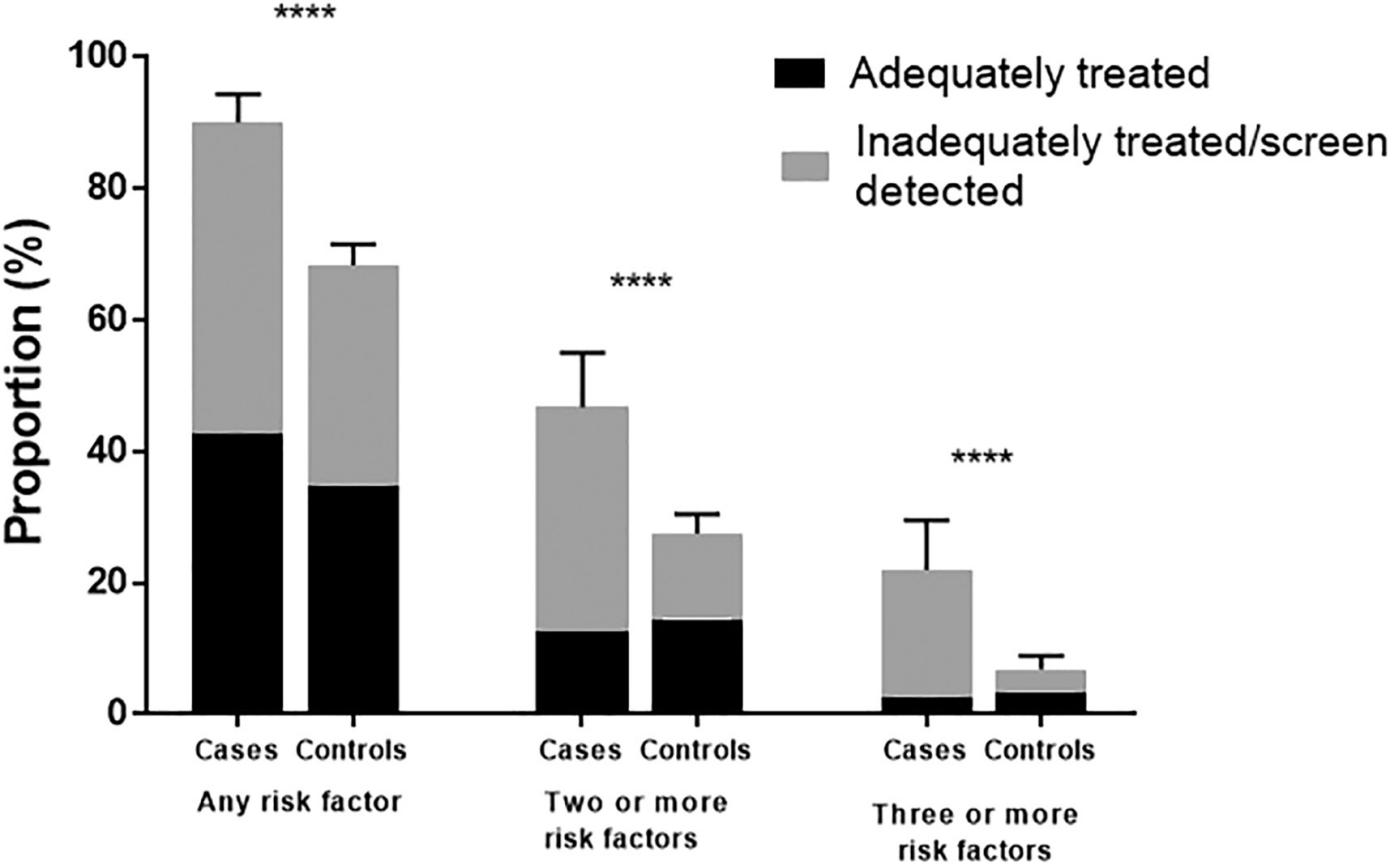
Proportion of cases and controls with one or more adequately treated or inadequately treated/screen detected cardiovascular risk factors. There was no significant difference in the prevalence of adequately treated risk factors between women with endometrial cancer and those without. The difference in the proportion of women with one, two or three or more cardiovascular risk factors between the two groups was thus due to the higher prevalence of previously undiagnosed and inadequately treated risk factors in women undergoing primary treatment for endometrial cancer.

Overall, a similar proportion of cases and controls were found to have at least one cardiovascular risk factor that had been previously diagnosed (42.7% cases vs. 34.9% controls, p = 0.08, fig. 3). The marked difference, however, was in the true underlying prevalence of these risk factors in the two populations. Significantly more women with endometrial cancer were found to have screen-detected or undertreated cardiovascular risk factors (88.7% cases vs 54.3% controls, p < 0.0001) and approximately a fifth were found to have at least three of these risk factors, which were not being adequately treated (19.3% cases vs. 3.5% controls, p < 0.0001).

### 3.3 Predicted 10-year cardiovascular disease risk in cases and controls

We calculated the QRISK2 score for cases and controls to determine the proportion of women at high risk who would benefit from primary cardiovascular disease prevention. As the QRISK2 score is only valid for patients without a history of cardiovascular disease, type I diabetes, familial hyperlipidemia and aged between 25 and 85 years, 11 cases and 124 controls were excluded from the analysis, leaving 139 cases and 622 controls for whom a QRISK2 score could be calculated. The resulting groups remained well matched for age and ethnic background and continued to demonstrate significant differences in the prevalence of overall, screen detected and undertreated cardiovascular risk factors whilst having similar levels of known risk factors (Supplementary Fig. 1a and b).

Two thirds of women with endometrial cancer compared with under half of controls met the National Institute of Clinical Excellence (NICE) threshold of a 10-year cardiovascular disease risk of 10% or greater for the introduction of statin therapy (63.3% cases vs. 46.6% controls, p = 0.0005, Table 2). Almost a third of cases had a QRISK2 score of 20% or greater, identifying them as being at high risk of cardiovascular disease in the next 10 years (29.5% cases vs. 16.9% controls, p = 0.001). The higher predicted cardiovascular disease risk in women with endometrial cancer compared with the general population was reflected in their higher median QRISK2 scores (12.6% in cases vs. 8.8% in controls, p < 0.0001).

**Table 2:** Predicted 10-year cardiovascular risk using QRISK2 score.

10 year cardiovascular risk	Cases (n = 139)	Controls (n = 622)	p value
< 10%, n (%)	51 (36.7)	332 (53.4)	0.0005⁎⁎⁎
≥ 10%, n (%)	88 (63.3)	290 (46.6)	
≥ 20%, n (%)	41 (29.5)	105 (16.9)	0.001⁎⁎⁎
Median (IQR) before risk factor optimization	12.6% (6.6–21.4%)	8.8% (3.5–17.1%)	< 0.0001⁎⁎⁎⁎
Median (IQR) after risk factor optimization	11.6% (6–18.9%)	8.4% (3.2–15.9%)	0.0004⁎⁎⁎
Absolute percentage change in cardiovascular risk following optimization	− 1.82	− 0.69	
Estimated number needed to treat to prevent one cardiovascular event over 10 years	55	145	

⁎⁎⁎ p < 0.001.

⁎⁎⁎⁎ p < 0.0001.

### 3.4 Estimated effect of risk factor optimisation

Finally, we estimated the likely benefit to arise from a screening program aimed at diagnosing and optimizing treatment of cardiovascular risk factors in endometrial cancer survivors. Interventions to promote weight loss, aiming for a BMI of 25 kg/m^2^, smoking cessation and optimization of hypertension and hypercholesterolemia treatment were shown to result in an absolute percentage reduction in cardiovascular risk of 1.8% for women with endometrial cancer compared with a reduction of 0.7% if undertaken in the control population. This equates to the treatment of 55 women with endometrial cancer to prevent one cardiovascular event (heart attack, transient ischemic attack or cerebrovascular accident) in the next 10 years. In contrast, 145 women in the general population would need to receive treatment to observe the same effect.

## 4 Discussion

In this study, the prevalence of obesity, diabetes, non-diabetic hyperglycemia, hypertension and hypercholesterolemia was significantly higher in women diagnosed with endometrial cancer than the general population. Almost all of the women with endometrial cancer had more than one risk factor for cardiovascular disease, with 22% having three or more concurrent risk factors. The true prevalence of these conditions, however, only becomes obvious when non-selective screening is performed, as endometrial cancer patients were much more likely to have cardiovascular risk factors that had not been detected and treated in primary care. Even when recognized, lipid and blood pressure control was frequently suboptimal. As a result, the women with endometrial cancer in this study had a 1.5-fold higher 10-year risk of cardiovascular disease, as measured using the QRISK2 score, compared with the general population. Many of these risk factors are modifiable and with optimization this absolute risk could be reduced by up to 1.8%, although it is likely to remain, on average, higher than for women without endometrial cancer. This is related to the fact that many endometrial cancer patients have multiple cardiovascular risk factors. Introduction of screening and treatment of cardiovascular risk factors in women following primary treatment for endometrial cancer would be predicted to be more effective than a similar program aimed at the general population, which is already advocated by NICE for people aged over 40 years [16].

Being diagnosed with cancer is a highly emotive experience that inevitably leads to questions about etiology, risk factors and prevention strategies. Some studies have shown that this ‘teachable moment’ is an opportunity for the successful introduction of lifestyle changes that improve overall survival [23]. Cardiovascular risk factor optimization could form part of that discussion; indeed, this is arguably more important than the efforts made in routine follow up to identify recurrent disease in women who have generally been cured of their endometrial cancer [24]. Weight loss, with its favorable impact on insulin resistance, blood pressure and cholesterol profiles, particularly given its low cost, low risk of harm, and added benefits for quality of life is the obvious answer [25], [26], but one that is very difficult to achieve and sustain by dietary restriction and lifestyle change [27]. A strategy of identifying and correcting hitherto unrecognized or undertreated cardiovascular risk factors with appropriate drug therapy may therefore offer a reasonable alternative for improving outcomes for endometrial cancer survivors. Bariatric surgery may also be appropriate for some women [28], [29].

### 4.1 Comparison with other studies

This is the first study to investigate the risk of non-fatal cardiovascular events in women with endometrial cancer. Few studies have previously measured the prevalence of individual risk factors for cardiovascular disease in this population, and they have often relied on self-reported co-morbidities or health records for known diagnoses only, and neither have they considered hypercholesterolemia in their assessment [15], [30]. The high prevalence of obesity [31] and diabetes [32] is well documented and a few studies have reported the burden of unrecognized insulin resistance and non-diabetic hyperglycemia in endometrial cancer patients [13], [33]. When 99 women with newly diagnosed endometrial cancer underwent screening with fasting serum glucose, 30.3% were found to have physician diagnosed diabetes and a further 36% were noted to have previously unrecognized insulin resistance [13]. These results are similar to our own, where 54.4% of women with a history of endometrial cancer were found to have non-diabetic hyperglycemia, although only 17.3% of women in our study had overt diabetes. This difference may be explained by differences in ethnicity and patients being part of distinct healthcare systems with differing rates of opportunistic screening.

Felix, Bower [8] found that deaths from cardiovascular disease were significantly more prevalent in women with a history of endometrial cancer in the Surveillance, Epidemiology and End Results Program (SEER) than in the general population. These results were not replicated in the Iowa Women's Health Study, though, where endometrial cancer survivors were noted to have a 25% lower risk of cardiovascular disease mortality compared with age and BMI matched women without a history of the disease [32]. The latter study was reliant on information recorded on death certificates to determine disease specific mortality rates and thus vulnerable to the inherent inaccuracies of these type of data. Cases were not only leaner than those in our study, with a median BMI of 28 kg/m^2^, but they were also BMI-matched to the controls. This eliminates the impact of obesity on other cardiovascular risk factors, all of which are strongly correlated. As part of a longitudinal study of lifestyle factors on cancer incidence, it is possible that participation in the study led to positive behavior change in women who developed endometrial cancer, reducing their subsequent risk of cardiovascular disease. This may also explain why there was no difference in the rates of non-fatal cardiovascular disease events in women with and without a history of endometrial cancer enrolled in the Women's Health Initiative [34]. As with the Iowa Women's Health Study, participants were healthier, with a lower prevalence of obesity and hypertension than in our study, potentially as a result of the ‘healthy bias’ associated with selective recruitment of women into clinical trials.

### 4.2 Strengths and limitations

The present study recorded known cardiovascular risk factors but additionally screened for asymptomatic, previously unidentified and inadequately treated hyperglycemia, hypertension and hypercholesterolemia to provide accurate prevalence data and a reliable estimation of 10-year cardiovascular disease risk in women undergoing primary treatment for endometrial cancer. The women enrolled in the Health Survey for England are highly representative of the general population, being selected at random on the basis of postcode rather than relying on self-recruitment into a study, which is known to introduce ‘healthy control’ bias. This makes them a reliable control group for comparison with the endometrial cancer cases. The study was also adequately powered to detect any differences in cardiovascular disease risk between the two populations, even after exclusion of individuals with a known history of cardiovascular disease and those not suitable for assessment using the QRISK2 score.

There were insufficient data contained within the Health Survey for England database to accurately determine whether individuals had a history of malignancy and of endometrial cancer in particular. This may have resulted in the misclassification of cases as controls, but would not have impacted upon the conclusions reached as indeed it would have biased results toward the null. This is even more likely given the limits imposed by the QRISK2 calculator with regards to extremes of body mass. As the model has only been validated for use in individuals with a BMI between 20 and 40 kg/m^2^, values outside of these are automatically replaced with the limit figure. Given the high prevalence of extreme obesity in the endometrial cancer group, this is likely to result in an underestimate of their cardiovascular disease risk and hence the benefit that may be derived from introducing screening and treatment for such risk factors. In addition, there was a paucity of individual level data in the Survey on the presence of renal disease, atrial fibrillation and rheumatoid arthritis, meaning that they could not be included as variables in the QRISK2 score for either group. However, this potential limitation is unlikely to have a significant impact on the final scores because the prevalence of these conditions is low in the general population. Whilst the QRISK2 score has only been validated in the UK population, the variables used to derive risk estimates are the same as those used in other risk calculators, including the Framingham cardiovascular risk calculator. Similar results would be expected if other risk calculators had been used.

### 4.3 Future work

Our data support the routine screening of women newly diagnosed with endometrial cancer for cardiovascular risk factors. We advocate measuring BMI, blood pressure, HbA1C and serum lipids with a view to calculating an individual woman's risk of cardiovascular disease using a validated risk prediction model, like QRISK2. Cardiovascular risk calculators could be added to the SGO Obesity Toolkit [35] to remind physicians to consider long term health issues for obese endometrial cancer patients, with prompts embedded in electronic patient records. Women at high risk of cardiovascular disease should be supported to reduce their risk through healthy lifestyle change to achieve weight loss and appropriate drug treatment to normalize their blood pressure, blood sugar and lipid levels. All women with a QRISK2 score ≥ 10% should be commenced on a statin, regardless of serum cholesterol [16]. Future research questions should focus on determining the impact of systematic screening and optimization of cardiovascular risk factors on cardiovascular event frequency as well as the optimal management strategy. Of particular interest is whether drug therapy for individual risk factors is superior to weight loss, achieved through dietary modification or bariatric surgery, for improving outcomes in endometrial cancer survivors [36].

## 5 Conclusions

Women undergoing primary treatment for endometrial cancer have a high prevalence of unrecognized and undertreated cardiovascular risk factors. Screening for and optimization of these conditions could favorably impact on future cardiovascular event frequency and improve overall survival in this population.

The following are the supplementary data related to this article.

**Supplementary Fig. 1a:**
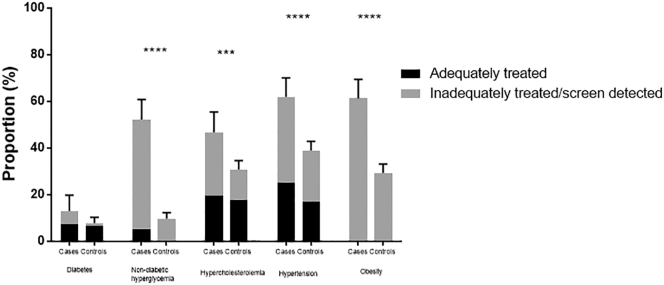
Prevalence of individual cardiovascular risk factors in cases and controls included in the QRISK2 analysis. In accordance with the results from the whole population, the significantly higher prevalence of cardiovascular risk factors in women with endometrial cancer was due to a greater proportion of women having previously unrecognized or inadequately treated conditions. The prevalence of adequately treated risk factors was similar between the two groups. The exception to this was diabetes, which affected a similar proportion of women with and without endometrial cancer (12.9% vs. 7.8%, p = 0.068).

**Supplementary Fig. 1b:**
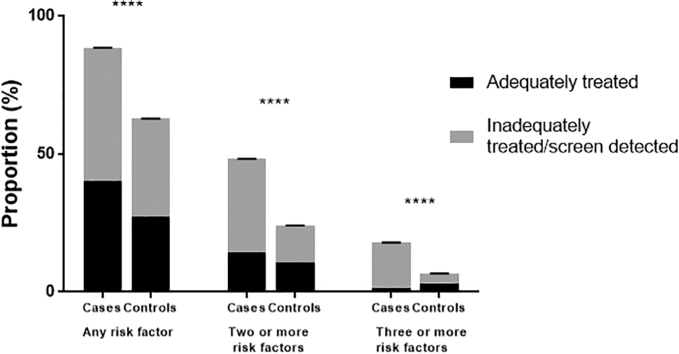
Prevalence of one or more cardiovascular risk factors. These results mirror those from the whole population analysis; significantly more women with endometrial cancer had at least one cardiovascular risk factor, with the majority being screen-detected or inadequately treated. At least one cardiovascular risk factor was present in 88.4% of endometrial cancer patients compared with 62.8% of the general population (p < 0.0001).

## Trial registration

NIHR Clinical Research Network Portfolio Study Identifier 30,602-Metabolic syndrome prevalence in endometrial cancer. The study was prospectively registered before data acquisition. The protocol originally stated that a diagnosis of metabolic syndrome would be used as a surrogate marker of cardiovascular risk. After taking advice from experts in the field, however, the QRISK2 score was substituted as it was deemed a more accurate and reliable measure of cardiovascular risk. This amendment was performed prior to data analysis.

## Details of contributors

SK, MR and EC designed the study. SK performed data collection and analyses and drafted the manuscript. JL, VS, ML and NR contributed to data collection. RE, ML, MR and EC contributed to data analysis. MR and EC contributed to the first draft of the manuscript. All authors contributed to the final manuscript. SK and EC had access to the data and can take responsibility for the integrity of the data and the accuracy of the data analysis.

## Competing interests

All authors declare: no support from any organisation for the submitted work; no financial relationships with any organisations that might have an interest in the submitted work in the previous three years; no other relationships or activities that could appear to have influenced the submitted work.

## Funding

EC and SK are funded through a National Institute for Health Research (NIHR) Clinician Scientist Fellowship (NIHR-CS-012-009). NR is an MRC Doctoral Research Fellow (MR/M018431/1). VS is funded by Wellbeing of Women and the Wellcome Trust (098670/Z/12/Z). This article presents independent research funded by the NIHR. The views expressed are those of the authors and not necessarily those of the NHS, the NIHR or the Department of Health. The funders had no involvement in study design, data collection, analysis and interpretation and in the decision to submit the article for publication.
